# Community pharmacists’ perceptions about pharmaceutical service of over-the-counter traditional Chinese medicine: a survey study in Harbin of China

**DOI:** 10.1186/s12906-016-1532-z

**Published:** 2017-01-05

**Authors:** Menghuan Song, Carolina Oi Lam Ung, Vivian Wing-yan Lee, Yuanjia Hu, Jing Zhao, Peng Li, Hao Hu

**Affiliations:** 1 State Key Laboratory of Quality Research in Chinese Medicine, Institute of Chinese Medical Sciences, University of Macau, Macao, China; 2 School of Pharmacy, Faculty of Medicine, Chinese University of Hong Kong, Hong Kong, China

**Keywords:** Community pharmacist, Community pharmacy, Over-the-counter, Pharmaceutical service, Traditional Chinese medicine, TCM, China

## Abstract

**Background:**

This study aims to investigate community pharmacist’s perception on the provision of over-the-counter (OTC) traditional Chinese medicine (TCM) pharmaceutical services; focusing on the areas of their attitude, general practice, perceived barriers and suggested improvements.

**Methods:**

Questionnaire survey targeting community pharmacists in Harbin of China was applied in this study. Questionnaires were distributed and collected at community pharmacies. Data was analyzed by combining descriptive analysis and Chi-test.

**Results:**

280 valid questionnaires were collected, giving a response rate of 78%. Respondents generally showed positive attitude towards OTC TCM pharmaceutical services. However, they were uncertain about whether such pharmaceutical services should be considered as their primary responsibility. Respondents indicated that they acted proactively to find out all the medicines taken by their patients and to remind consumers of possible OTC TCM adverse reactions. However, they were less keen on recommending or re-directing consumers to suitable OTC TCM. The three main barriers hindering the provision of OTC TCM pharmaceutical service identified in this study were “insufficient professional knowledge” (54.6%), “ambiguity of the professional role of pharmacists” (54.6%) and “lack of scientific evidence of OTC TCM” (45.4%). The three main actions considered most relevant to improving pharmaceutical service of OTC TCM were “formulating or refining legislation to clarify the legal and professional role of pharmacists with respect to TCM” (60.7%), “strengthening training of pharmacists with respect to TCM” (57.9%), and “promoting public awareness of the pharmacist’s role” (53.6%). According to the results of Chi-test, respondents’ perceptions about the attitude, practice, perceived barriers, and improvement suggestions were significantly different depending on the education levels, certificate types and workloads of western medicine.

**Conclusions:**

The community pharmacists in Harbin, China were positive about the provision of OTC TCM pharmaceutical services. However, they were less certain about taking this duty as their primary responsibility. Insufficient knowledge and lack of role definition in the area of OTC TCM were found to be the major factors discouraging the provision of pharmaceutical service on OTC TCM by community pharmacists.

**Electronic supplementary material:**

The online version of this article (doi:10.1186/s12906-016-1532-z) contains supplementary material, which is available to authorized users.

## Background

Traditional Chinese medicine (TCM), especially over-the-counter (OTC) TCM, are often taken for self-medication without the advice of pharmacists or physicians [1]. The perceived safety of TCM contributes to the wide acceptance of OTC TCM and results in a misled perception that these products are without risks [2]. Lack of professional supervision may expose consumers to various risks [3]. Incidents of adulteration of TCM with active pharmaceutical ingredients, poor product quality, side effects and drug interactions have been reported [4]. The increased risks of side effects and interactions associated with concurrent use with western medicines (WM) may also be neglected [5].

Pharmaceutical service provided by pharmacists helps to ensure that drug therapy is provided to achieve definite outcomes for the improvement of patient’s quality of life [6]. It is considered one of the key practice to alleviate the concerns over drug safety for consumers*.* However, application of pharmaceutical service in the area of TCM and other modalities of traditional medicine (TM) is not clearly determined and so are the legal and ethical responsibilities, competencies and levels of practice standards of pharmacists with respect to TM. According to previous studies, the role of pharmacists about TM was ambiguous in Sudan, Canada and Hong Kong [7–9]. Pharmacists in Saudi Arabia and Singapore were shown to have shortage of knowledge with respect to TM [10, 11]. Some studies identified limited resources and insufficient professional knowledge as the major barriers to the provision of professional service related to TM or complementary medicine by pharmacists in Argentina, Japan and Australia [12–14].

In China, the health care system reform launched in 2009 emphasized the importance of primary health care through which secure, efficient, convenient and affordable health care services should be provided with full coverage (http://www.wpro.who.int/china/mediacentre/factsheets/health_sector_reform/en/). Accordingly, pharmacists in community pharmacies have an important role to play as a gate keeper at the community level in drug safety and minor health problems by providing pharmaceutical service. Unlike many countries where the health care service is dominated by WM, the two streams of health care services, TCM and WM, are practiced alongside each other in China, where TCM receives strong support from government policies [15]. Therefore, the need for developing pharmaceutical service on TCM, WM and integrative medicine is prominent. However, the concept of pharmaceutical service was only introduced in China in 1990s and the development has been slow especially at community level compared to hospital settings. A study found that there was a general lack of high-quality counselling services and a shortage of qualified pharmacists to meet increasing patient needs, resulting in a low level of pharmaceutical service delivery [16].

The way that pharmacists practice in China also raises concerns. According to the ‘Licensed Pharmacist Examination Implementation Measures’ issued by the China Food and Drug Administration (CFDA), there are two types of pharmacists recognized legally: TCM pharmacists and general pharmacists and TCM pharmacists are allowed to practice only in TCM whereas general pharmacists only in WM [17]. However, in practice, it is a common phenomenon that the sales and counselling of TCM is handled by either general pharmacists or TCM pharmacists in community pharmacies. Despite the high usage and growing importance of TCM in China, no research has been conducted to specifically assess the quality of TCM pharmaceutical service provided by community pharmacists.

This study aimed to investigate community pharmacist’s perception on the provision of over-the-counter (OTC) traditional Chinese medicine (TCM) pharmaceutical services; focusing on the areas of their attitude, general practice, perceived barriers and suggested improvements. This study also explores the potential differences between general pharmacists and TCM pharmacists in the community. The findings are expected to give references for improving pharmaceutical service of TM not only in China but also in other countries where there is an increasing role of community pharmacists in the provision of TM products.

## Methods

### Data collections

A written questionnaire survey method was applied in this study. This research design was reviewed and approved by the Ethics Committee of the University of Macau.

Harbin City, the capital of Heilongjiang Province in the north of China, was chosen as the research site for several reasons. Firstly, it has a long history of providing pharmaceutical service. In 1902, the first community pharmacy, Songhua River Pharmacy, was founded by Russian, which enabled increased accessibility of both TCM and WM. Secondly, there is a large population in Harbin with a considerable number of community pharmacies. In 2012, the population of Harbin reached about 372 million. In the same year, there were 2152 community pharmacies in Heilongjiang Province, of which 70% were in Harbin [18]. The findings of this study in Harbin are therefore expected to offer valuable insights.

In order to determine the sample size, three steps were taken. Firstly, the official website of Harbin Food and Drug Administration was consulted to obtain a list of 1506 community pharmacies registered in Harbin and their locations. Secondly, to accurately locate the pharmacies geographically and to reconfirm the number of pharmacies, information on the official website was compared against what was shown on the Internet map (map.baidu.com). Considering the 9 municipal districts in Harbin, stratified sampling based on these districts was applied to ensure that findings of this study would sufficiently represent all social and economic demographics. The pharmacies in each municipal district were then coded. Thirdly, to determine the sample size for the 1506 community pharmacies in Harbin, the margin of error (standard error) of 0.05 with 95% confidence interval was used and the calculated sample size was 307 pharmacies. Considering that all questionnaires would be personally distributed and collected by researchers on-site, and the experience of the researchers, a non-response rate was expected to be small. An additional contingency of 10 questionnaires for non-response and inappropriate responses were used to result in a final sample size of 317 pharmacies, giving a sampling ratio of 21% (317/1506). Using 21% as the sampling ratio, 21% of the pharmacies in each municipal district were selected randomly.

The investigation was completed in December in 2014. The targets of the investigation were all the pharmacists in community pharmacies. Pharmacists were identified by inquiry or badge observation. To initiate the investigation, researchers first introduced themselves and then explained the background and the purpose of the study to the pharmacists according to the research introduction pages of the questionnaire. Respondents were also told that the study was anonymous and would not harm their personal interests. Finally, researchers obtained respondents’ consent before commencing the questionnaire. Researchers waited on-site while respondents completed the questionnaire without intervention. When respondents raised queries, researchers responded objectively and only mentioned relevant considerations. Questionnaires were immediately collected upon completion.

### Measurement

The original survey questionnaire was developed based on thorough review of past literature [19, 20]. The questionnaire was originally written in English based on literature review as shown in Additional file 1. It was then translated to Chinese and underwent several revisions based on feedbacks from pharmacists who were fluent in both English and Chinese. This was to ensure the use of the most appropriate wordings for the validity of the questionnaire and thus greater acceptance and understanding by Chinese pharmacists participated in this study. Pilot survey was conducted in 58 community pharmacies in Zhuhai City (a different city) to test the validity of the questionnaire. Necessary modifications were made based on the comments received from the pilot survey (see Additional file 1).

The questionnaire comprised of three parts. The first part entailed the community pharmacist’s personal information including gender, age, seniority, education level, business type of pharmacy (franchising vs. independent), type of registration certificate (general pharmacist vs. TCM pharmacist), and WM workload. Seniority was used to determine respondents’ experience as a pharmacist. Considering the fact that general pharmacists may also practice TCM, respondents were asked about their type of registration certificate. WM workload measured the workload percentage of a pharmacist’s practice related to WM.

The second part explored respondents’ perceptions concerning OTC TCM pharmaceutical services. Two statements were used to measure their attitude towards the provision of OTC TCM pharmaceutical services. (1 = completely disagree; 5 = completely agree). There were another 4 statements designed to measure the extent of OTC TCM pharmaceutical services provided by pharmacists to consumers (1 = never; 5 = To all consumers). In addition to the above, multiple-choice questions of six-items were used to study the respondents’ perceptions of barriers hindering the provision of OTC TCM pharmaceutical services. Finally, multiple-choice questions of seven-items were used to collect respondents’ suggestions to improving OTC TCM pharmaceutical services.

### Data analysis

Data analysis was conducted by combining descriptive analysis and Chi-test. The background information of respondents, their attitude, practice, and perceived barriers and suggestions were firstly analyzed in a descriptive way. In addition, Chi-test was applied to assess the possible relationships among respondent’s answers (about attitude, practice and perceived barriers) with their gender, age, seniority, education level, type of pharmacy, type of registration certificate, and WM workload. Statistics significance of *P* < 0.05 was defined. All the data were input and analyzed with SPSS 20.0 for Windows.

## Results

### Background information of respondents

317 community pharmacies where a total of 360 pharmacists worked were selected as the distribution target of the questionnaire. 360 questionnaires were distributed to the pharmacists. Among them, 43 pharmacists refused to participate and 317 pharmacists participated and returned the answered questionnaire. Out of the 317 questionnaires returned, 37 were omitted due to incomplete answer or obvious response errors. 280 completed questionnaires were considered valid for further analysis, giving a response rate of 78%.

As shown in Table 1, 72.5% respondents were female and 51.8% respondents worked at franchising pharmacies. The majority was aged between 20 to 40 years. Most respondents’ seniority was less than 10 years of which 31.8% respondents only worked as pharmacist for less than 5 years and 46.1% had seniority of 5–10 years.

**Table 1:** Background information of respondents (*N* = 280)

Item	n (%)
Gender	
Male	77 (27.5)
Female	203 (72.5)
Age	
20-30	82 (29.3)
30-40	150 (53.6)
40-50	38 (13.6)
50-60	8 (2.9)
≥ 60	2 (0.7)
Seniority	
< 5 years	89 (31.8)
5-10 years	129 (46.1)
10-20years	53 (18.9)
≥ 20 years	9 (3.2)
Education	
College and below	183 (65.4)
Bachelor	81 (28.9)
Master	16 (5.7)
Pharmacy business type	
Franchising pharmacy	145 (51.8)
Independent pharmacy	135 (48.2)
Certificate type	
General pharmacist	157 (56.1)
TCM pharmacist	123 (43.9)
WM workload	
> 0%& < 20%	35 (12.5)
20%-40%	53 (18.9)
40%-60%	52 (18.6)
60%-80%	78 (27.9)
80%-100%	62 (22.1)

The respondents’ education level was relatively low: 65.4% respondents’ education background was only college level or below; and 28.9% had bachelor degree. 56.1% of the respondents were certificated as general pharmacist. Half of the respondents had a WM workload range of 60% -100% while 20% had a WM workload range of 40%-60% and 30% had less than 40% WM workload.

By using Chi-test, it was found that respondents’ education level, certificate type and WM workload had significant effects on their perceptions. All the other demographic factors of respondents had no significant impact.

### Respondents’ attitude towards pharmaceutical service of OTC OTM

As shown in Table 2, 66.8% respondents agreed or completely agreed that they would do their best to provide OTC TCM pharmaceutical service. Nevertheless, only 45.4% respondents agreed or completely agreed that their primary responsibility was to provide OTC TCM pharmaceutical service and 45% respondents disagreed or completely disagreed that their primary responsibility was to provide OTC TCM pharmaceutical service.

**Table 2:** Respondents’ attitude towards pharmaceutical service (*N* = 280)

	n (%)					Educ-ation	Certif-icate	WM workl-oad
	Completely disagree	Disagree	Not sure	Agree	Completely agree	*P*	*P*	*P*
I would do my best to provide OTC TCM pharmaceutical service	8 (2.9)	43 (15.4)	42 (15.0)	154 (55.0)	33 (11.8)	.002^*^	.000^*^	.001^*^
My primary responsibility is to provide OTC TCM pharmaceutical service	26 (9.3)	100 (35.7)	27 (9.6)	106 (37.9)	21 (7.5)	.001^*^	.000^*^	.000^*^

^*^ *P* <0.05

By using Chi-test, it was found that respondents’ attitude was significantly different among education level, certificate type and WM workload. The pharmacists with college degree or below showed more positive attitude. Comparatively, the pharmacists with bachelor degree or above showed more negative attitude. For pharmacist certificate type, general pharmacists were negative about their primary responsibility of providing OTC TCM pharmaceutical service while TCM pharmacists were shown to be more positive.

### Respondents’ practice towards pharmaceutical service of OTC TCM

As shown in Table 3, about 30% respondents would find out all the medicines taken by patients when necessary. Over 40% respondents chose “as frequent as possible” or “to all consumers”, indicating a proactive manner. Also, about 30% respondents would remind consumers of possible OTC TCM adverse reactions when necessary. Additionally, 44% respondents chose “as frequent as possible” or “to all consumers”, inferring active practice towards OTC TCM pharmaceutical service.

**Table 3:** Respondents’ practice towards pharmaceutical service (*N* = 280)

	n(%)					Educ-ation	Certif-icate	WM workl-oad
	Never	Only upon consum-ers’ request-s	Whene-ver necessa-ry	As frequ-ent as possi-ble	To all consu-mers	*P*	*P*	*P*
How often would you find out all the medicines taken by patients?	21 (7.5)	61 (21.8)	77 (27.5)	71 (25.4)	50 (17.9)	.000^*^	.000^*^	.000^*^
How often would you recommend suitable OTC TCM to consumers?	15 (5.4)	79 (28.2)	95 (33.9)	58 (20.7)	33 (11.8)	.057	.000^*^	.018^*^
How often would you re-direct consumers to the right OTC TCM?	17 (6.1)	75 (26.8)	84 (30.0)	60 (21.4)	44 (15.7)	.002^*^	.000^*^	.006^*^
How often would you remind consumers of possible OTC TCM adverse reactions?	18 (6.4)	60 (21.4)	79 (28.2)	67 (23.9)	56 (20.0)	.002^*^	.000^*^	.009^*^

^*^ *P* <0.05

On the other hand, pharmacists did not show a strong intention of recommending OTC TCM or re-directing to the right OTC TCM with about 30% respondents chose “when necessary”, about 30% chose “never” or “only upon consumers’ requests”, and about 30% respondents chose “as frequent as possible” or “to all consumers”.

The results of Chi-test showed that respondents’ practice was significantly different among education level, certificate type and WM workload. Specifically, general pharmacists showed passive practice when providing OTC TCM pharmaceutical service and TCM pharmacists showed more proactive approach. For WM workload, the respondents with less than 20% WM workload showed passive practice when providing OTC TCM pharmaceutical service. However, the respondents with more than 20% WM workload showed active intention to provide this service.

### Respondents’ perception of about barriers

As shown in Table 4, respondents considered that the three main barriers hindering the OTC TCM pharmaceutical service were “insufficient professional knowledge” (54.6%), “ambiguity of the professional role of pharmacists” (54.6%) and “lack of scientific evidence of OTC TCM” (45.4%).

**Table 4:** Barriers to provide pharmaceutical service at community pharmacy (*N* = 280)

Barriers items	n (%)
Insufficient professional knowledge	153(54.6)
Ambiguity of the professional role of pharmacists	153(54.6)
Lack of scientific evidence of OTC TCM	127(45.4)
Unwillingness of consumers with respect to the OTC TCM service	111(39.6)
Lack of time	76(27.1)
Lack of reimbursement	60(21.4)

Table 5 showed that the respondents’ perceptions about the three main barriers were significantly different among education level, certificate type and WM workload. Particularly, respondents with bachelor degree or above considered “ambiguity of the professional role of pharmacists” as a major barrier. Besides, respondents with less than 60% WM workload recognized “ambiguity of the professional role of pharmacists” as an important barrier when providing OTC TCM pharmaceutical service.

**Table 5:** Chi-test for top 3 barriers

	Education	Certificate type	WM workload
	*P*	*P*	*P*
Insufficient professional knowledge	.921	.228	.000^*^
Ambiguity of the professional role of pharmacists	.002^*^	.000^*^	.000^*^
Lack of scientific evidence of OTC TCM	.011^*^	.717	.309

^*^ *P* < 0.05

### Respondents’ improvement suggestions

As shown in Table 6, the three most important actions suggested by the respondents to improve OTC TCM pharmaceutical service were “formulating or refining legislation to clarify the legal professional role of pharmacists with respect to TCM” (60.7%), “strengthening training of pharmacists with respect to TCM” (57.9%), and “promoting public awareness of the pharmacist’s role” (53.6%).

**Table 6:** Suggestions for improving pharmaceutical service at community pharmacy (*N* = 280)

Improvement suggestions	n(%)
Formulating or refining legislation to clarify the legal and professional role of pharmacists with respect to TCM	170(60.7)
Strengthening training of pharmacists with respect to TCM	162(57.9)
Promoting public awareness of the pharmacist’s role	150(53.6)
Expanding the access to the information of evidence-based TCM	149(53.2)
Formulating or refining the standards of pharmacists’ practice with respect to TCM	140(50.0)
Providing enough professionals to ensure the quality of TCM pharmaceutical service	108(38.6)
Providing reasonable reimbursement to pharmacists	54(19.3)

Table 7 showed that the three main suggestions chosen by respondents were significantly different among education level, certificate type and WM workload. In particular, respondents with bachelor degree or above preferred to consider “promoting public awareness of the pharmacist’s role” as an important improvement suggestion. General pharmacists and respondents with less than 60% WM workload agreed more on “promoting public awareness of the pharmacist’s role” and “strengthening training of pharmacists with respect to TCM” as main improvement suggestions.

**Table 7:** Chi-test for top 3 suggestions

	Education	Certificate type	WM workload
	*P*	*P*	*P*
Promoting public awareness of the pharmacist’s role	0.000^*^	0.000^*^	0.000^*^
Strengthening training of pharmacists with respect to TCM	0.093	0.000^*^	0.000^*^
Formulating or refining legislation to clarify the legal and professional role of pharmacists with respect to TCM	0.008^*^	0.176	0.162

^*^ *P* < 0.05

## Discussion

This research investigated community pharmacist’s perceptions on the provision of OTC TCM pharmaceutical services in Harbin, China which raised some important issues worth further discussion. Pharmacists in this study were positive about providing OTC TCM pharmaceutical services. This was probably due to the need evident in the customers and the potential drug risks associated with un-supervised use of TCM. The positive attitude to adopt such role might also be due to the fact that it is required by law. This finding corresponds to the results of another study in which 63% pharmacists were found to have positive attitude towards pharmaceutical service with respect to complementary and alternative medicine (CAM) [11]. A growing body of evidence suggests that practitioners including pharmacists are willing to discuss and incorporate complementary and alternative medicine into their practice [21–23]. Nevertheless, although most of the pharmacists in this study would like to do their best to provide OTC TCM pharmaceutical service, less than half of them agreed or completely agreed that their primary responsibility was to provide OTC TCM pharmaceutical service. This indicated that safe and appropriate use of OTC TCM was not considered as their priority by many pharmacists. Pharmacists’ professional role in the area of OTC TCM was not prominent.

The three main barriers hindering the OTC TCM pharmaceutical services identified in this study were “insufficient professional knowledge”, “ambiguity of the professional role of pharmacists” and “lack of scientific evidence of OTC TCM”. Similar findings have been reported in previous studies [12, 24–28]. Insufficient knowledge was the most reported barrier. A study carried out in the U.S. reported that 44% of participating pharmacists acknowledged that their knowledge of herbs and natural products was inadequate [29]. Studies in Australia, the United Kingdom, the U.S. and Singapore indicated that pharmacists rated their knowledge and skills to counsel patients on CAMs as inadequate [11, 30–33]. The increased use of TCM including herbal and complementary medicines, particularly herbal remedies and dietary supplements, warrants the need for pharmacists to have basic knowledge of the products and keep up to date with the current development in this area [34].

More than half of the respondents agreed that unclear professional role would hinder the OTC TCM pharmaceutical service. The role of pharmacist refers to the overall duty of care that pharmacists should and can provide for the general public and patients. As the development of pharmaceutical services in China is only at its early stages when compared to more advanced countries, the general public and even the pharmacists themselves may not have a thorough understanding of what pharmacists should offer professionally [35, 36]. Pharmacists often experience difficulties in accessing integrated, non-biased and evidence-based information, especially when many community pharmacists do not have access to TCM information resources, which is also a common barrier observed in other countries [14, 37]. The results of this study also showed that pharmacists considered the lack of evidence-based medicine (EBM) to support the use of OTC TCM as one of the major barriers to OTC TCM pharmaceutical service. Although many pharmacists did not consider that the shortage of EBM in TCM would necessarily mean the lack of safety and efficacy, the quest of EBM in TCM would continue to grow unless other measures became available for pharmacists to use for evaluating the safety and efficacy of TCM [38].

The barriers identified in this study appeared to reflect similar experience in other countries. However, on a closer look to the Chi-test results, there were major contributing factors to these barriers specific to the pharmaceutical service system in China that intensified the complexity of improving the situation: pharmacists’ education level, certificate type and workload of WM. These underlying causes posed significant impact on pharmacists’ perceptions and practice. The nature and intensity of basic education varied greatly among practicing pharmacists in China. Many of them only received secondary school or college education due to the lack of a systematic bachelor degree of pharmacy in the past. To sit for the qualification examination and to become a licensed pharmacist, one could have education specialized in pharmacy or related disciplines. Applicants with secondary school education, college education, bachelor degree and master degree could apply for the examination after working for seven, five, three and one year in the pharmacy area respectively. No working experience was required for applicants with PhD degree [16, 17]. Despite the lack of standardized education qualification, they were allowed to become licensed pharmacists and provide pharmaceutical service in community pharmacies, like any other formally trained community pharmacists, once they passed the examination.

The Chi-test results also showed a significant difference between general pharmacists and TCM pharmacists in the level of agreement/disagreement about their role in OTC TCM and how they practiced. Nearly 75% of the TCM pharmacists completely agreed or agreed that providing OTC TCM pharmaceutical service was their primary responsibility. However, over 68% of the general pharmacists completely disagreed or disagreed on this matter. More than half of the TCM pharmacists would recommend suitable OTC TCM to all consumers or as frequently as possible. Over 60% TCM pharmacists would re-direct to the right OTC TCM for all consumers or as frequently as possible. However, less than 20% general pharmacists would recommend suitable OTC TCM to all consumers or as frequently as possible. Only about 15% general pharmacists would re-direct to the right OTC TCM for all consumers or as frequently as possible. The difference in the perceptions about their professional role and the way each type of pharmacists practice towards TCM was a reflection of how these two types of pharmacists were trained and regulated in the pharmaceutical service system in China.

According to the legislation, the pharmaceutical service system separated the practice of general pharmacists and TCM pharmacists on either WM or TCM exclusively in the same way that pharmacists in other countries such as Canada, Singapore and Australia would provide service on WM only [24]. General pharmacists were not expected to be involved in the supply and counseling of OTC TCM, which explained why pharmacists did not recognize TCM as their core responsibility and thus the lack of motivation in their practice as observed in this study. The separation of practice mandated by the pharmaceutical system has the benefit of providing more focused professional service. Nevertheless, both TCM pharmacists and general pharmacists were found to provide pharmaceutical service on OTC TCM in practice.

Furthermore, the separation of practice also contributed to diversification of pharmacy education into TCM and WM, which was shown to significantly affect pharmacists’ belief and practice towards OTC TCM. The knowledge requirements for general pharmacists and TCM pharmacists were made specific to each modality. As far as qualifying examinations are concerned, candidates for licensed TCM pharmacists are required to have adequate TCM-related knowledge whereas general pharmacists are required to acquire adequate knowledge about WM only. In addition, TCM teaching is currently not standardized and might not be included in the syllabus of pharmacy training for general pharmacists. It is the lack of knowledge about TCM among general pharmacists which resulted in their different ways of practice. As a result, their level of competency varied and pharmaceutical service was practiced to various extents.

This is an important observation reflecting the deviation of practical situation from the legal perspectives which should prompt careful consideration especially by the competent authority. CFDA might need to evaluate the practical situations and strengthen regulations to ensure general pharmacists and TCM pharmacists practice within their scope of practice in a way that can fully address the needs of the general public. At the moment, general pharmacists might have crossed the line in terms of their scope of practice to provide service on OTC TCM when they could be ill-equipped with necessary TCM knowledge. In light of integrative medicine, however, isolated practice of pharmacists within the domains of TCM and WM may no longer be ideal especially when considering drug-related problems associated with concurrent use of TCM and WM. There might be a need for a bridging role between the two streams of pharmaceutical products and pharmacists should be encouraged to provide necessary interventions to ensure safety and appropriateness of the concurrent use of WM and TCM. In order to improve and ensure the quality of pharmaceutical service of OTC TCM provided by pharmacists at community level, external force like government is crucial in formulating or refining legislation to clarify the legal and professional role of pharmacists based on the practical situation and the actual needs of the consumers [39]. Government should also help to promote the corresponding roles of the two types of pharmacists to the general public. With the aid of government, community pharmacies should also take the initiative to promote their roles to the public.

To address the problem of knowledge insufficiency, there should be measures targeting pharmacy students as well as practicing pharmacists. Schools and colleges in the U.S. have already considered the demand for formal education and training of herbal and natural products, and reviewed the training course for pharmacists accordingly [40]. In particular, educational strategies and programs have been developed to teach practicing pharmacists and pharmacy students how to support consumers in making appropriate self-care choices [41, 42]. Taking into account the separation of TCM and WM practice in the pharmaceutical service system, universities and institutions in China should consider incorporate more TCM related teaching to address the arising safety issues associated with the integration of TCM. For this, training of the basic concept and philosophy of TCM is important as a starting point.

For practicing pharmacists, adequate professional resources such as continuous training opportunities should be in place to support their performance. Frequent exposure to continuing education of TCM helped ensure pharmacists to be knowledgeable about TCM [34]. The American Pharmacists Association (APhA) has developed the DrugInfoLine in order to help pharmacists keep pace with the updated health supplement knowledge [43]. Continuous education on TCM should be emphasized and formal courses on TCM should be made available to pharmacists to help ensure the maintenance and improvement of their professional knowledge. On the other hand, reliable sources of information with regards to the safe use of TCM should be made accessible at the workplace for health care professionals to help patients make informed choices and avoid drug-related problems [11]. On this matter, the involvement of pharmacist professional organizations is critical.

In China, policy that emphasizes on the importance of community pharmacists came into effect in recent years to give a solid foundation for the development of pharmacist’s professional role at community level. One of the most important professional bodies representing pharmacists is the Chinese Pharmaceutical Association (CPA). As a regulatory and professional body for pharmacists and pharmaceutical scientists, CPA is entrusted with the responsibility to define pharmacists’ professional role and to provide them with support to advance their level of pharmaceutical service. CPA is also looked upon to play an active role in working with the government, universities and community pharmacies to establish standard of practice, guidelines and regulations to develop and promote pharmaceutical service of OTC TCM provided by community pharmacists. Continuous education and training activities organized by CPA and other professional bodies are also considered essential for capacity building to prepare community pharmacists with sufficient knowledge and skills. At the moment, clear guidance and adequate support from professional bodies to encourage the provision of pharmaceutical service about OTC TCM seemed to be lacking and immediate actions by professional bodies such as CPA are very much anticipated.

### Limitation

This study has two limitations that could be addressed in the future study. Firstly, this research targeted Harbin as research site, which provided empirical evidence that was rarely reported in the past literature. Future study in other areas would provide more information about pharmaceutical service of community pharmacist in China. Secondly, while this research reported information from the view of community pharmacists, future research from the perspective of consumer would provide more detailed information about the demand for OTC TCM pharmaceutical service in China.

## Conclusion

The current study confirmed the interests of community pharmacists in Harbin City of China to provide best OTC TCM pharmaceutical service. However, the ambiguity of defined roles of general pharmacists and TCM pharmacists as well as the lack of OTC TCM knowledge posed great barriers for the provision of quality professional service. The profound implications of capacity building and role development for pharmacists to take on the role in the area of OTC TCM prompted the need for a collaborative effort from the authority, universities and professional organizations.

## Additional file

Additional file 1: Questionnaire. (DOCX 24 kb)

## Abbreviations

APhA: American Pharmacists Association
CAM: Complementary and alternative medicine
CFDA: China Food and Drug Administration
OTC: Over-the-counter
TCM: Traditional Chinese medicine
WM: Western medicine
