# Supplementary material for: Community pharmacists’ perceptions about pharmaceutical service of over-the-counter traditional Chinese medicine: a survey study in Harbin of China
Source: BMC Complement Altern Med. 2017 Jan 5;17:9. doi: 10.1186/s12906-016-1532-z (PMC5217429; doi:10.1186/s12906-016-1532-z)
Supplement: Additional file 1: — Questionnaire. (DOCX 24 kb) [file 12906_2016_1532_MOESM1_ESM.docx]

**Additional files:**

**Additional file 1: Questionnaire**

**Community pharmacists’ perceptions about pharmaceutical care of over-the-counter traditional Chinese medicine: a survey study in Harbin of China**

Dear Madam/Sir,

We are from the Institute of Chinese Medical Sciences, University of Macau. We are now carrying out a study on **Community pharmacists’ perceptions about pharmaceutical care of over-the-counter traditional Chinese medicine: a survey study in Harbin of China**. The purpose of this research is to investigate community pharmacists’ standpoint and their viewpoint for the question “Should pharmacists be considered one of the key practice to safeguard drug safety of OTC traditional Chinese medicine for the public?” Your contribution is important for the success of this research. For this, we would like to ask your kind cooperation to complete the survey.

This survey is solely for academic research purposes. It does not involve any commercial interests. Information collected will remain anonymous and not be revealed to any third-party. Confidentiality will be assured for personal and public interests.

Your contribution to this research is highly appreciated.

Thank you very much.

**Background and definition of important terms**

**1. Background**

Traditional Chinese medicine (TCM), especially over-the-counter (OTC) TCM, are often taken for self-medication without the advice of pharmacists or physicians. This lack of professional supervision may expose the consumer to various risks. Pharmaceutical care provided by pharmacists has been considered as one of the key practice to alleviate the problem. However, different from many other countries, OTC TCM pharmaceutical care is provided by both licensed general pharmacists and TCM pharmacists in China. Therefore, this study aims to investigate community pharmacist’s perceptions about pharmaceutical care of OTC TCM from the aspects of attitude, practice, perceived barriers, and improvement suggestions.

**2. Definition of important terms**

Three important concepts are clarified in order to facilitate your understanding of our investigation.

**Traditional Chinese medicine (TCM)**: In China, TCM cover a wide of items including traditional Chinese medicinal materials, Chinese herbal medicine, Traditional OTC Chinese medicine and national medicine (such as Mongolian medicine, Tibetan medicine, Uygur medicine, Dai medicine).

**Over-the-counter drug (OTC)**: Prescription drugs and over-the counter drugs with safety and effectiveness are two kinds of drugs approved by the China Food and Drug Administration (CFDA). Over-the-counter (OTC) drugs could be purchased and used by self-judgment without a prescription.

**Self-medication**: Self-medication is a [human behavior](http://en.wikipedia.org/wiki/Human_behavior) in which an individual chooses and uses a medicinal product with recognized safety and efficacy available over-the-counter to treat self-determined disease or symptoms.

The drugs referred in this study are all over-the-counter (OTC) drugs.

| **Community pharmacists’ attitude, practice and perceived barriers about pharmaceutical care of OTC TCM** |
| --- |
| **The First Part. Demographics of pharmacists** |
| - 1. Sex ❒ Male ❒ Female   2. Age ❒20-30 ❒30-40 ❒40-50 ❒50-60 ❒≥60   3. Seniority ❒＜5 years ❒5-10 years ❒10-20 years ❒≥20 years   4. Education ❒ College and below ❒ Bachelor ❒ Master ❒ PhD   5. Pharmacy type ❒ Franchising pharmacy ❒ Independent pharmacy (max.3 branches)   6. Certification type ❒General pharmacy ❒TCM pharmacy   1.7 Western medicine workload ❒0％ ❒>0%&<20% ❒20％－40% ❒40%－60%  ❒60％-80％ ❒80%－100% ❒100% |
| **The Second Part. Community pharmacists’ attitude, practice and perceived barriers about pharmaceutical care of OTC TCM** |
| 1. C**ommunity pharmacists’ attitude about pharmaceutical care of OTC TCM (single-answer question)** |
| 1.1 I would do my best to provide OTC TCM pharmaceutical service  ❒ Strongly Disagree ❒ Disagree ❒ Neutral ❒ Agree ❒ Strongly Agree  1.2 My primary responsibility is to provide OTC TCM pharmaceutical service.  ❒ Strongly Disagree ❒ Disagree ❒ Neutral ❒ Agree ❒ Strongly Agree |
| 2. C**ommunity pharmacists’ practice about pharmaceutical care of OTC TCM (single-answer question)** |
| 2.1 How often would you find out all the medicine taken by patients?  ❒ Never ❒ Only upon customers’ requests ❒ Whenever necessary  ❒As frequent as possible ❒ To all consumers  2.2 How often would you recommend suitable OTC TCM to consumers?  ❒ Never ❒ Only upon customers’ requests ❒ Whenever necessary  ❒As frequent as possible ❒ To all customers  2.3 How often would you re-direct customers of right OTC traditional Chinese medication?  ❒ Never ❒ Only upon customers’ requests ❒ Whenever necessary  ❒As frequent as possible ❒ To all customers    2.4 How often would you remind consumers of possible OTC TCM adverse reactions?  ❒ Never ❒ Only upon customers’ requests ❒ Whenever necessary  ❒As frequent as possible ❒ To all customers |
| **3. Community pharmacists’ perceived barriers and improvement advice about pharmaceutical care of OTC TCM (multiple-answer question)** |
| 3.1 What do you think are the barriers of pharmacists providing pharmaceutical care in relation to OTC TCM?  ❒ Shortage of professional knowledge  ❒Ambiguity of the professional role of pharmacist  ❒Unwillingness of customers with respect to the OTC TCM service  ❒Lack of scientific evidence of OTC TCM  ❒Lack of time  ❒Lack of remuneration  ❒Others（please specify）：__    3.2 What do you think are the most important improvement measures from the list below towards the provision of pharmaceutical care in relation to OTC TCM?  ❒ Promoting public education of pharmacist role  ❒Strengthen the training of pharmacists with respect to TCM  ❒Formulating or refining legislation to clarify the legal and professional role of pharmacists with respect to TCM  ❒ Formulating or refining the standards of pharmacists’ practice with respect to TCM  ❒ Expanding the access to the information of evidence-based TCM  ❒ Providing enough professionals to ensure the quality of TCM pharmaceutical service  ❒ Providing reasonable reimbursement to pharmacists  ❒Others（please specify）：_______________________________ |
